# New Insights Into Causal Pathways Between the Pediatric Age-Related Physical Activity Decline and Loss of Control Eating: A Narrative Review and Proposed Conceptual Model

**DOI:** 10.3389/fpsyg.2020.578690

**Published:** 2020-10-14

**Authors:** Tyler B. Mason, Kathryn E. Smith, Britni R. Belcher, Genevieve F. Dunton, Shan Luo

**Affiliations:** ^1^Department of Preventive Medicine, University of Southern California, Los Angeles, CA, United States; ^2^Department of Psychiatry and Behavioral Science, University of Southern California, Los Angeles, CA, United States; ^3^Department of Psychology, University of Southern California, Los Angeles, CA, United States; ^4^Department of Medicine, University of Southern California, Los Angeles, CA, United States

**Keywords:** loss of control eating, physical activity, executive functioning, inhibitory control, pediatrics

## Abstract

Research consistently suggests that loss of control (LOC) eating in children and adolescents is a key factor contributing to pediatric obesity and eating disorders. However, causes of pediatric LOC eating are yet unclear, and there is a lack of longitudinal research investigating the developmental processes contributing to LOC eating and related outcomes in youth. Physical activity is an understudied behavior that declines during middle childhood to adolescence and may exert an influence in the development of LOC eating *via* its impact on executive functioning. While physical activity levels and executive functioning have been linked to regulation of eating, no research has examined the mechanistic processes by which these domains may together impact LOC eating during childhood and adolescence. In the current narrative review, a model is proposed that suggests how physical activity and executive functioning influence LOC eating and related outcomes during childhood and adolescence. This model has the potential to influence future theoretical models of pediatric LOC eating and guide future prevention and intervention efforts.

## Introduction

As children transition from childhood to adolescence, they become increasingly responsible for their own eating behavior – including types of food eaten, how much food is eaten, and when they eat (Bassett et al., 2008). In the obesogenic food environments that are omnipresent in modern society, ability to self-regulate eating and control responses to food are critical for prevention of negative pediatric health outcomes including obesity, type 2 diabetes, and eating disorders. Loss of control (LOC) eating is a behavior that is characterized by a subjective sense of LOC over what or how much one is eating (irrespective of quantity consumed) and is associated with elevated caloric intake particularly from snacking and intake of energy-dense foods (e.g., fast food and sweets) as well as eating disorder pathology and mood and anxiety disorders (Goldschmidt et al., 2017).

LOC eating begins to emerge across middle childhood into adolescence, with recent data showing that up to 30% of children and adolescents with overweight or obesity report LOC eating with similar prevalence across sex (He et al., 2017). Importantly, children who report LOC eating are more likely to gain weight over time and develop full syndrome eating disorders and/or mood and anxiety disorders (Goldschmidt, 2017; Byrne et al., 2019). Specifically, previous data show that LOC eating predicts sub‐ or full-threshold binge-eating disorder diagnosis and greater global eating disorder psychopathology (Tanofsky-Kraff et al., 2011; Hilbert et al., 2013). Despite the prognostic relevance of LOC eating for longer-term psychological and physical health, the etiology and maintenance of LOC eating in youth remains poorly understood, as predominant theoretical models of disordered eating (e.g., affect regulation and interpersonal models) have not held up consistently in children and adolescents (Hilbert et al., 2009; Ranzenhofer et al., 2014; Goldschmidt et al., 2018b). This is a crucial problem for prevention and intervention efforts, which is further evidenced by the limited efficacy of existing weight management and eating disorder interventions in children.

## Physical Activity Decline in Middle Childhood and Adolescence

Alongside observed increases in LOC eating during middle childhood and adolescence, there is a well-documented age-related decline in physical activity levels as children enter middle childhood and puberty, such that only 24.8% of youth ages 12–15 meet physical activity guidelines of daily moderate-to-vigorous physical activity for at least 60 min (Fakhouri et al., 2014). This decline is not well-understood, but may be driven by biological factors (Belcher et al., 2013; Spruijt-Metz et al., 2013), environmental and psychosocial factors (Sallis et al., 2000), or decreases in participation in organized sports (Perez et al., 2017; Kemp et al., 2019). In addition to decreasing physical activity, sedentary behaviors increase during adolescence. Taken together, middle childhood through adolescence are critical years during which LOC eating develops and physical activity levels are simultaneously declining.

## Physical Activity and Pediatric LOC Eating

Although physical activity and LOC eating share intriguingly similar developmental timeframes during which significant changes in these behaviors occur, they are almost entirely studied apart from one another. Consistently, recent reviews of the literature on LOC eating in youth did not discuss physical activity as relevant risk factor for LOC eating (Byrne et al., 2019; Tanofsky-Kraff et al., 2020). Nevertheless, mounting evidence suggests that higher overall physical activity may exert beneficial effects on eating behavior, and this is supported by studies in adults showing that higher levels of physical activity are related to better appetite regulation, reduced food cue responsivity, and less binge eating (Joseph et al., 2011; Luo et al., 2018). Similarly, among children and adolescents, higher accelerometer-assessed physical activity was negatively correlated with naturalistically-assessed LOC eating, overeating, stress‐ and emotion-related eating, and hunger (Smith et al., 2020a,b). Given such data, physical activity has been termed a “gateway behavior” that may facilitate improvements in related health behaviors, including eating. These findings are especially relevant for youth with overweight or obesity given that LOC eating and physical inactivity are more prevalent in this group compared to peers of lower weight (Harriger and Thompson, 2012; Prentice-Dunn and Prentice-Dunn, 2012; He et al., 2017). Thus, it is possible that higher physical activity levels could have beneficial effects on eating patterns that in turn mitigate poor long-term outcomes among children.

Importantly, the influence of physical activity on eating may occur both at the momentary level (e.g., minutes to hours) and over extended time periods (e.g., months to years). At the momentary level, in children and adults, acute bouts of activity have been shown to attenuate appetite and urges to consume palatable food and have been linked to decreases in energy intake in children and adults (Thayer et al., 1993; Maraki et al., 2005; Taylor and Oliver, 2009; Thivel and Chaput, 2014). Further, prior naturalistic research among adults with obesity found that dietary lapses and temptations were less likely to occur after exercising (Carels et al., 2004). Also, elevated momentary moderate-to-vigorous physical activity predicted less stress-related eating in adolescents with higher BMI-z and predicted less positive emotional eating in adolescents with lower BMI-z (Smith et al., 2020b). Conversely, physical inactivity may have detrimental short-term effects on eating regulation. While directionality cannot be inferred, an ecological momentary assessment (EMA) study of high school adolescents found that consumption of sweet snacks was concurrently associated with sedentary activities such as watching television and using electronic media at the same prompt (Grenard et al., 2013).

In addition to these momentary associations, longitudinal research in adults has shown that adults participating in exercise interventions experience greater increases in healthy eating patterns (i.e., increased fruit and vegetable intake and decreased junk food consumption) relative to non-intervention conditions (Oaten and Cheng, 2006; Fleig et al., 2011). Among adults with overweight and obesity, higher lifestyle physical activity, measured at the end of a 12-month behavioral weight loss program, was also related to greater flexible dietary restraint, less disinhibited eating, and less perceived hunger at 12‐ and 36‐ month follow-up assessments (Carraça et al., 2013). In sum, there is evidence that physical activity may have both short‐ and long-term beneficial effects on eating. However, there remains a dearth of literature that has examined such relationships in children and adolescents, particularly with respect to key behaviors (i.e., LOC eating) that are linked to current and future physical and mental health problems.

## Executive Functioning as a Mechanism Linking Activity and LOC Eating

Moreover, the mechanisms underlying associations between physical activity and eating regulation have yet to be elucidated. While several factors have been posited to contribute to these relationships, burgeoning evidence indicates acute and long-term physical activity behavior enhance executive functioning, and poor executive functioning increases risk for the development of LOC eating (Verburgh et al., 2014; Goldschmidt et al., 2015; Alvarez-Bueno et al., 2017; Tanofsky-Kraff et al., 2020). Executive functions refer to “top-down” cognitive processes that guide goal-directed behavior and allow for adaptations to changing circumstances, and which are rooted in circuitry within the prefrontal cortex (Diamond, 2013). These executive functions develop throughout adolescence and are critically important for adaptive self-regulatory processes, including eating and physical activity behaviors (Hofmann et al., 2012; Dohle et al., 2018). In particular, inhibitory control deficits (i.e., reduced ability to suppress or interrupt prepotent responses) can interfere with self-regulation processes, including the ability to modulate the types and amount of food consumed (Liang et al., 2014) and are a specific facet of executive functioning that may be related to LOC eating. In fact, a recent study found that inhibitory control deficits, assessed with the stop-signal task, were the only executive functioning measure associated with caloric consumption during a laboratory test meal – an objective measure of LOC eating (Kelly et al., 2020).

While studies of inhibitory control in children and adolescents have most commonly utilized self-report measures and behavioral tasks, cognitive neuroscience research has begun identifying brain pathways associated with inhibitory control deficits. Neuroimaging studies using functional magnetic resonance imaging (fMRI) to examine inhibitory control reliably implicate frontostriatal circuitry, including areas of the lateral prefrontal cortex (Dias et al., 1997; Aron et al., 2004). In adolescents with obesity, disinhibited eating has been linked to reduced integrity of frontal lobe, specifically lower orbitofrontal cortex volume (Maayan et al., 2011). Another recent study found that after completion of a food-specific inhibitory control task, overweight adolescents showed reduced activation in frontal inhibitory regions, including the superior frontal gyrus, middle frontal gyrus, ventrolateral prefrontal cortex, medial prefrontal cortex, and orbitofrontal cortex, compared to adolescents of lower weight (Batterink et al., 2010). Thus, it appears that less activation in the prefrontal cortex, important for inhibition control when trying to inhibit response to palatable food, is associated with greater weight and as an extension more dysregulated eating behaviors, such as LOC eating.

In addition to inhibitory control predicting LOC eating, research suggests that physical activity improves inhibitory control in children and adolescents. Several studies of children or adolescents found that physical activity was associated with acute improvements in inhibitory control (Chang et al., 2014; Browne et al., 2016; Franco-Alvarenga et al., 2019). Further, adolescents who completed an 8-week exercise program had increased inhibitory control following the intervention compared to a control group (Ludyga et al., 2018). While cognitive neuroscience research is limited (Belcher et al., 2020), one study found that physical activity may enhance prefrontal cortex functioning in children (Chaddock-Heyman et al., 2013). In addition, in a separate fMRI study, children with higher fitness level had more efficient brain networks associated with inhibitory control compared to children with lower fitness level (Voss et al., 2011).

## Model of Physical Activity Decline, Executive Functioning, and Pediatric LOC Eating

**Figure 1:**
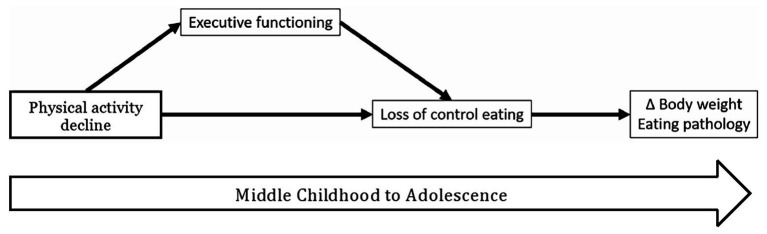
Model of activity, executive functioning, loss of control eating, and long-term outcomes.

Given current limitations, we introduce a hypothesized model (Figure 1) that posits that developmental changes in physical activity patterns and executive functioning together influence self-regulation from childhood into adolescence, such that declines in physical activity have negative short‐ and long-term effects on behavioral and neural markers of executive functioning, which in turn increases risk for subsequent LOC eating and poor health outcomes (e.g., obesity and eating disorder pathology) over time. While there is likely a bi-directional relationship between executive functioning and physical activity such that executive functioning also precipitates regular physical activity, there is a more substantial literature on the predictive association from physical activity to executive functioning in children (Hillman et al., 2011; Verburgh et al., 2014; de Greeff et al., 2018). In addition, studies using accelerometers to obtain objective measurements of physical activity have shown that children who engaged in more physical activity had better executive functioning (Syväoja et al., 2014; van der Niet et al., 2015).

Physical activity has also been shown to alter neurobiological processes associated with inhibitory control. For example, children with overweight and obesity who underwent a 3-month aerobic exercise intervention, compared to a control group, evidenced increased recruitment of the bilateral prefrontal cortex during an inhibitory control (i.e., antisaccade) task that was completed at baseline and post-intervention (Davis et al., 2011). Further, physical activity has been shown to be particularly effective at improving executive functioning in children with obesity (Logan et al., 2020), whom are more at-risk for LOC eating (He et al., 2017).

The proposed model has not been extensively studied and stems from connecting the available literatures on physical activity and LOC eating in children and adults. Investigation of this model has several potential theoretical and clinical implications. Research has implicated a number of factors in relation to LOC eating in children; however, little is known about the developmental origins of LOC eating (Byrne et al., 2019). That is, much more is known about how children who exhibit LOC eating differ from children who do not exhibit LOC eating opposed to the mechanisms that explain the initial onset of LOC eating. Although, research has yet to study differences in physical activity between children with vs. without LOC eating. One of the risk factors associated with LOC eating in children is impairments in general and food-specific aspects of executive functioning (Allen et al., 2013; Goldschmidt et al., 2015; Goldschmidt et al., 2018a; Stojek et al., 2018; Van Malderen et al., 2018). This proposed model is the first to suggest that a decline in physical activity that occur in middle childhood may be a biobehavioral mechanism that explains the onset of LOC eating in childhood.

In addition to theoretical implications, the proposed biobehavioral model could have high clinical significance. There have been limited studies investigating treatments for LOC eating in children. Those that have been conducted have studied psychological therapies (e.g., cognitive-behavioral therapy) as a treatment for LOC eating and have reported these therapies to be successful (Byrne et al., 2019). Examples of possible clinical implications of our theoretical model might be using physical activity as a stand-alone intervention or as part of psychotherapy to treat LOC eating in children. However, empirical research will be needed to determine appropriate clinical recommendations – including types of activity, duration, and frequency that are needed to change executive functioning and behavior. Further, from a preventive standpoint, while we know that it is crucial for children to remain physically active throughout childhood and adolescence to reduce negative physical and psychological outcomes, testing of the hypothesized model can provide information about physical activity as a preventive measure for LOC eating.

This model also may inform the combination and sequencing of prevention and intervention components, particularly if strategies that promote physical activity exert a transfer effect on eating regulation *via* enhancing executive functions. Furthermore, consistent with precision medicine initiatives, analysis of momentary, real-time data will be crucially important to inform tailored treatments. New preventions or treatments could target certain *types* of children or traits (e.g., children high vs. low in inhibitory control) or target the specific *moments* at which a child is most prone to engage in LOC eating (e.g., states of physical inactivity and reduced inhibitory control). Further, it is critical for pediatricians to screen for children’s adherence to physical activity recommendations in early childhood and utilize behavior change techniques with children and parents to increase adherence.

It is important to acknowledge limitations and other considerations. The key limitation of this review and proposed conceptual model is that it is based on a small number of studies, and we draw on some studies from the adult literature given the comparatively sparse pediatric literature base. In addition, this is a proposed conceptual model that is intended to guide further research direction and has not yet been tested, and thus, empirical research will be needed in order to make clinical recommendations. The model described is intentionally parsimonious to guide initial research in this area, yet there is a plethora of other variables that should be considered in the context of this model moving forward. For example, emotion regulation is an important factor related to physical activity and LOC eating (Goldschmidt et al., 2017; Bernstein and McNally, 2018), and emotion regulation abilities are modulated by executive functioning (Calkins and Marcovitch, 2010; Sudikoff et al., 2015). Therefore, emotion regulation abilities likely play an important role in this model. Further, other trait and dispositional variables are key to examine as moderators and mediators within the context of this model including personality (e.g., health consciousness and impulsivity), familial factors (e.g., parenting practices), and environment (e.g., proximity to fast food outlets or parks).

Finally, while reviews of physical activity and executive functioning (Hillman et al., 2011; Verburgh et al., 2014; de Greeff et al., 2018) have all shown evidence for relationships between physical activity and executive functioning, there have been inconsistent findings regarding acute vs. chronic activity effects on executive functioning, depending upon measure used. Verburgh et al. (2014) concluded that acute physical activity (i.e., single bout of activity) predicted improved executive functioning using task-based measures but chronic physical activity (i.e., long-term exercise programs) did not; though, there were a limited number of chronic physical activity studies. Conversely, Hillman et al. (2011) reported that acute (i.e., single bout of activity) and chronic (i.e., fitness level) physical activity both predicted improved executive functioning using task and event-related potential measures. de Greeff et al. (2018) also found that acute (i.e., single bout of activity) and chronic (i.e., long-term exercise program) physical activity both predicted improved executive functioning using task measures, but results differed across tasks. These reviews demonstrate the importance for studying possible effects of both acute and chronic activity. Importantly, future studies testing this model should use objectively measured accelerometer physical activity, which measures children’s total volume of activity and can account for all activity that children perform.

In sum, establishing causal pathways and micro-temporal associations among physical activity, executive functioning, and LOC eating in youth has the potential to inform new prevention and intervention strategies for a host of pediatric outcomes. Future studies using multi-method designs, including psychological interviews, ambulatory assessment, and cognitive assessment, across middle childhood and adolescence will be needed to test the proposed model. Research on moderators and facets of executive functioning will also be needed to refine the model.

## Author Contributions

TM and KS: conceptualized the idea and wrote the first draft of the manuscript. BB, GD, and SL: revised subsequent drafts and provided critical feedback. All authors contributed to the article and approved the submitted version.

### Conflict of Interest

The authors declare that the research was conducted in the absence of any commercial or financial relationships that could be construed as a potential conflict of interest.
